# Folding and Binding Kinetics of the Tandem of SH2 Domains from SHP2

**DOI:** 10.3390/ijms25126566

**Published:** 2024-06-14

**Authors:** Livia Pagano, Valeria Pennacchietti, Francesca Malagrinò, Mariana Di Felice, Julian Toso, Elena Puglisi, Stefano Gianni, Angelo Toto

**Affiliations:** 1 Dipartimento di Scienze Biochimiche “A. Rossi Fanelli”, Laboratory Affiliated to Istituto Pasteur Italia-Fondazione Cenci Bolognetti, Sapienza Università di Roma, 00185 Rome, Italy; livia.pagano@uniroma1.it (L.P.); valeria.pennacchietti@uniroma1.it (V.P.); mariana.difelice@uniroma1.it (M.D.F.); julian.toso@uniroma1.it (J.T.); elena.puglisi@uniroma1.it (E.P.); stefano.gianni@uniroma1.it (S.G.); 2 Dipartimento di Medicina Clinica, Sanità Pubblica, Scienze della Vita e Dell’ambiente, Università dell’Aquila, Piazzale Salvatore Tommasi 1, Coppito, 67010 L’Aquila, Italy; francesca.malagrino@univaq.it

**Keywords:** protein–protein interactions, protein folding, protein domain, kinetics

## Abstract

The SH2 domains of SHP2 play a crucial role in determining the function of the SHP2 protein. While the folding and binding properties of the isolated NSH2 and CSH2 domains have been extensively studied, there is limited information about the tandem SH2 domains. This study aims to elucidate the folding and binding kinetics of the NSH2-CSH2 tandem domains of SHP2 through rapid kinetic experiments, complementing existing data on the isolated domains. The results indicate that while the domains generally fold and unfold independently, acidic pH conditions induce complex scenarios involving the formation of a misfolded intermediate. Furthermore, a comparison of the binding kinetics of isolated NSH2 and CSH2 domains with the NSH2-CSH2 tandem domains, using peptides that mimic specific portions of Gab2, suggests a dynamic interplay between NSH2 and CSH2 in binding Gab2 that modulate the microscopic association rate constant of the binding reaction. These findings, discussed in the context of previous research on the NSH2 and CSH2 domains, enhance our understanding of the function of the SH2 domain tandem of SHP2.

## 1. Introduction

Protein–protein interactions (PPIs) are at the basis of most fundamental biological processes, orchestrating the vast majority of physiological and molecular pathways crucial for cellular function and homeostasis [1,2,3,4,5]. Understanding the molecular determinants that underlie the mechanisms by how a specific protein–protein interaction domain recognizes and binds one (or more) specific ligand is essential for deciphering the intricacies of biological systems, as well as the pathological states that are rooted within them. Over the years, significant efforts have been made to characterize isolated domains in their folding mechanisms and binding reactions, overlooking them within the context of larger supramodular structures [6]. In fact, up to date, little information is available about the binding mechanisms of PPI domains within the framework of supramodular arrangements, including tandem repeats or multidomain architectures [7,8,9,10,11,12,13]. This approach is critical to pinpoint possible dynamic interplay occurring during the recognition and binding of ligands, such as allosteric, cooperative behaviors and complex intramolecular energetic networks that may regulate protein function and would allow us to better understand the role of those proteins in delivering cellular signaling networks.

**Figure 1:**
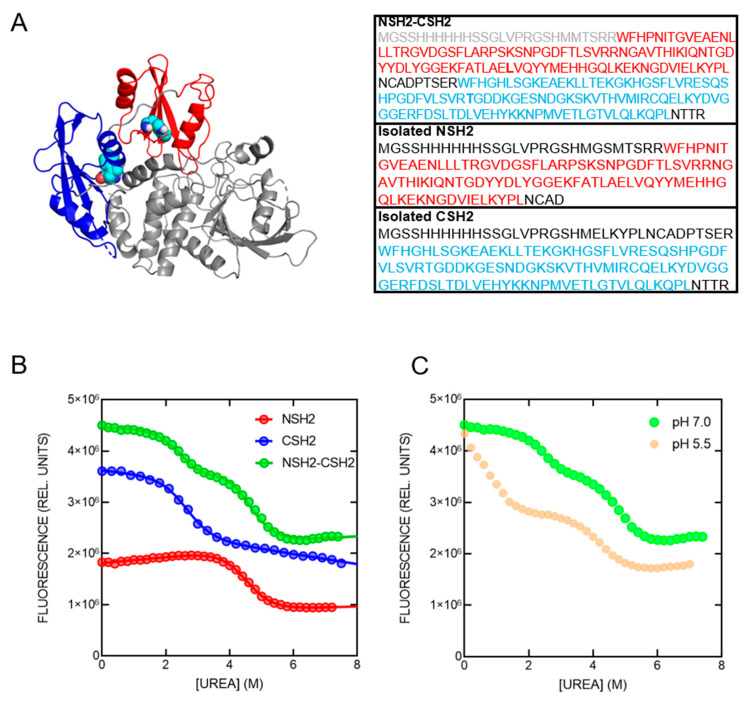
(**A**) Cartoon representation of the three-dimensional structure of the SHP2 protein (PDB: 2SHP). The NSH2 and CSH2 domains are colored in red and blue, respectively. The two tryptophan residues naturally present in the SH2 domains are highlighted as cyan spheres. The sequences of the constructs used in this study are also reported, following the same color code. (**B**) Equilibrium unfolding experiments conducted on the isolated NSH2 domain (in red) and CSH2 domain (in blue) and on the NSH2-CSH2 tandem (in green). Lines represent the best fit to a sigmoidal equation (for the isolated domains) and to the sum of two sigmoidal equations (for the tandem). Thermodynamic parameters calculated: NSH2 m_D-N_ = 1.4 ± 0.1 kcal mol^−1^ M^−1^, midpoint = 4.4 ± 0.1 M; CSH2 m_D-N_ = 1.4 ± 0.1 kcal mol^−1^ M^−1^, midpoint = 2.6 ± 0.1 M. The double sigmoidal equation used to fit the equilibrium denaturation of the tandem was forced to the parameters obtained for the isolated domains, returning an excellent fit (R^2^ = 0.98). (**C**) Equilibrium denaturation of the NSH2-CSH2 tandem at pH 7.0 (in green) and at pH 5.5 (in light orange). It is possible to observe that the first transition of the double sigmoidal curve appears dramatically shifted to lower urea concentrations, while the second transition is less affected by the change in pH.

SHP2 (Src Homology 2 domain-containing Phosphatase 2) is a pivotal signaling protein implicated in several cellular processes, including proliferation, differentiation, migration and apoptosis [14,15,16,17,18,19]. Notably, the three-dimensional structure of SHP2 is characterized by the presence of two tandemly arranged SH2 domains, named NSH2 and CSH2, that mediate the interaction of SHP2 with ligands presenting a phosphorylated tyrosine, and a PTP (Protein Tyrosine Phosphatase) domain that retains the catalytic activity of the protein (Figure 1A) [20]. When the protein is in its inactive state, the NSH2 domain physically interacts with the catalytic site of the PTP domain blocking it. Protein activity is triggered when the NSH2 domain binds a ligand, causing a conformational change of the domain that releases the catalytic site of the PTP domain which becomes able to dephosphorylate its substrates [20,21,22,23,24]. This mechanism of activation can occur through the binding of both mono- and the studied related ligands [25,26,27], although the binding with both SH2 domains provides a stronger activation and more specific binding. The CSH2 domain binds different portions of the same ligands recognized by the NSH2, thus playing an important role in orienting the ligand and cooperating with the NSH2 domain in the activation of the enzyme. The two domains display, in fact, different specificities in the recognition of residues flanking the phosphorylated tyrosine, with the CSH2 being more specific compared to the NSH2, which appears to be more promiscuous [28,29].

Due to the crucial importance of the binding event mediated by the SH2 domains of SHP2 in the regulation of several molecular pathways in the cell, the NSH2 and CSH2 domains have been extensively characterized. Studies on the SH2 tandem construct have been focused mainly on the structural orientation of the two domains during the binding event [30,31]. Recently, in a study conducted through a combination of molecular dynamics (MD) simulations, NMR and SAXS data highlighted a malleable structure of the NSH2-CSH2 tandem, with the NSH2 domain able to populate many different orientations in relation to the CSH2 [32]. On the other hand, the biophysical characterization of the binding and folding properties of the NSH2 and CSH2 domains has focused mainly on isolated constructs [33,34,35], and no information is currently available about the tandem SH2 domains.

Here, we aim to provide such information by employing folding and binding kinetic experiments of the NSH2-CSH2 tandem domains explored through fast kinetic experiments. Building upon previously obtained results on the isolated domains [33,34,35,36], we provide a comprehensive characterization of the folding mechanism of the NSH2-CSH2 tandem explored at different pH conditions. The analysis of data highlights that, while in most of the conditions explored, the two domains appear to fold and unfold independently one to the other, more complex scenarios arise at acidic pH. The comparison of the kinetic data obtained on the tandem construct with the ones obtained on the isolated domains suggests the accumulation of a misfolded intermediate at pH 5.5, resembling what was previously observed for other multidomain protein systems [37,38,39]. We also explored the binding kinetics of the NSH2-CSH2 tandem with peptides mimicking different portions of Gab2 protein, a physiological ligand of SHP2. The results obtained indicate that the NSH2 and CSH2 domains are selective for specific portions of Gab2. Intriguingly, in the context of the tandem construct, the affinity of NSH2 for Gab2 appears to be slightly lower when the CSH2 domain is bound to its ligand compared to when it is not, with an effect on the microscopic association rate constant. The results are discussed in light of the previous work on this protein system.

## 2. Results

### 2.1. The NSH2 and CSH2 Domains Appear to Fold and Unfold Independently of One Another

To characterize the folding mechanism of the NSH2-CSH2 tandem of SHP2, we conducted equilibrium and kinetic (un)folding experiments. Equilibrium urea-induced denaturation of the NSH2-CSH2 tandem was performed in buffer Hepes 50 mM, 2 mM DTT, pH 7.0 following the change in fluorescence of the two tryptophan residues naturally present in the construct, i.e., W6 and W112, respectively, on the NSH2 and the CSH2 domain. As reported in Figure 1B, the change in fluorescence at 340 nm is consistent with a double transition, which could be fitted with an equation consisting of the sum of two sigmoidal curves. The fluorescence emission of the native (0 M urea) and denatured protein (at 7.4 M urea) are reported in Figure S1. By comparing the equilibrium unfolding curve of the NSH2-CSH2 construct with those obtained for the isolated domains (data published in [34,35] and reported in Figure 1B), it is possible to observe that the equilibrium transition of the tandem is consistent with the sum of the thermodynamic parameters (m_D-N_ value and midpoint) obtained for the two isolated domains, forced in the equation of the double sigmoidal curve and returning an excellent fit. This congruence suggests that, within the NSH2-CSH2 tandem, the two domains undergo unfolding independently of one another, without discernible inter-domain interactions, unfolding without mutual influence. It is important to notice that decreasing the pH to acidic values (5.5) caused a dramatic shift towards lower urea concentrations of the first transition of the double sigmoidal curve, while the second transition remained mostly unaffected (Figure 1C). This suggests that the thermodynamic stability of the CSH2 domain appears to be more susceptible to changes in pH compared to the NSH2 domain.

**Figure 2:**
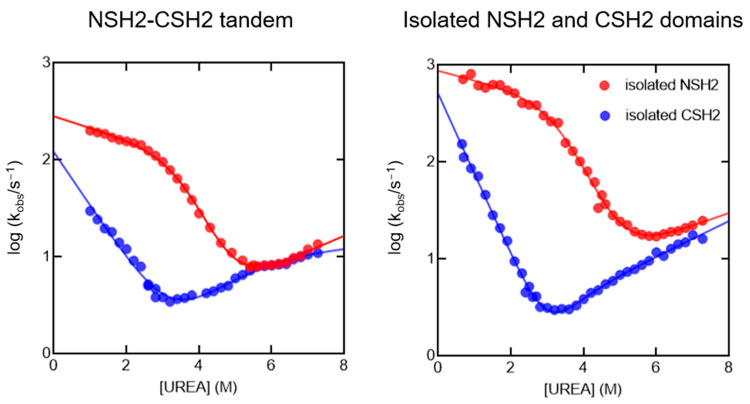
Kinetic (un)folding experiments conducted on the NSH2-CSH2 tandem in Hepes 50 mM, 2 mM DTT, pH 7.0 at 25 °C (left panel). We could record two phases by using two different optical filter, 320 nm cutoff (in red) and 360 nm cutoff (in blue). The comparison with the folding kinetics obtained for the two isolated domains at the same experimental conditions (right panel) highlights a high resemblance of the fast phase to the kinetics of the NSH2 domain (in red), and of the slow phase to the kinetics of the CSH2 domain (in blue).

To obtain information about the folding mechanism of the NSH2-CSH2 tandem, we performed unfolding and refolding kinetic experiments through stopped-flow methodology. Experiments were conducted in Hepes 50 mM, 2 mM DTT, pH 7.0, monitoring the change of tryptophan fluorescent emission as a function of time. At all the final urea concentrations explored, we could monitor the presence of two different kinetic phases by using different optical filters (320 nm cut-off and 360 nm cut-off). A plot reporting the logarithm of the observed rate constants as a function of urea concentrations (chevron plot) of the two visible phases, together with those obtained previously on the isolated domains at the same experimental conditions, is presented in Figure 2. Inspection of the kinetic data suggests that the two phases observed can be ascribed to the folding and unfolding of the NSH2 and CSH2 domains in the context of the tandem construct. These data suggest that, under these experimental conditions, the folding and unfolding processes of each domain proceed independently, with no apparent interactions between the two.

### 2.2. Effect of pH on the (Un)folding Kinetics of the NSH2-CHS2 Tandem

**Figure 3:**
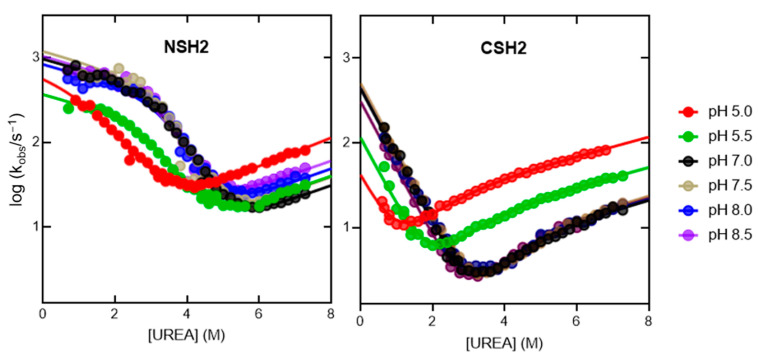
Folding kinetics of the isolated NSH2 (on the left) and CSH2 domain at different pH values (data from [34]). Chevron plots of the NSH2 domain were fitted to an equation implying the presence of an accumulating intermediate in rapid pre-equilibrium with the denatured state, while the chevron plots of the CSH2 domain were fitted to an equation that describes the curvature in the unfolding arm as caused by a change in rate-limiting step at different denaturant concentrations. Kinetic m values were shared for all datasets. NSH2—m_IN_ = 0.16 ± 0.02 kcal mol^−1^ M^−1^; m_DI_ = 1.0 ± 0.1 kcal mol^−1^ M^−1^; m_NI_ = 0.20 ± 0.02 kcal mol^−1^ M^−1^. CSH2—m_f_ = 1.14 ± 0.01 kcal mol^−1^ M^−1^; m_u_ = 0.73 ± 0.04 kcal mol^−1^ M^−1^; m_part_ = 0.56 ± 0.06 kcal mol^−1^ M^−1^.

To further characterize the folding mechanism of the NSH2-CHS2 tandem, we conducted kinetic folding and unfolding experiments in different experimental conditions, i.e., changing the pH of the solution. While for the isolated CSH2 domain, a complete analysis of the pH dependence of the folding kinetics is already available [34], no information is available on the effect of pH on the folding of the NSH2 domain. Thus, we performed a complete pH dependence of the kinetics of folding and unfolding of the NSH2 domain, and the results are shown in Figure 3. In accordance with previous works [35], kinetic data were fitted with a three-state equation, implying the accumulation of an intermediate along the reaction pathway. The resulting data are listed in Table S1. It is possible to observe that, while for the CSH2 domain the change towards acidic pH provokes a strong thermodynamic destabilization of the protein, the NSH2 domain appears to be less susceptible to pH changes, with its stability being mostly unaffected until pH 5.5, in agreement with equilibrium experiments reported above.

**Figure 4:**
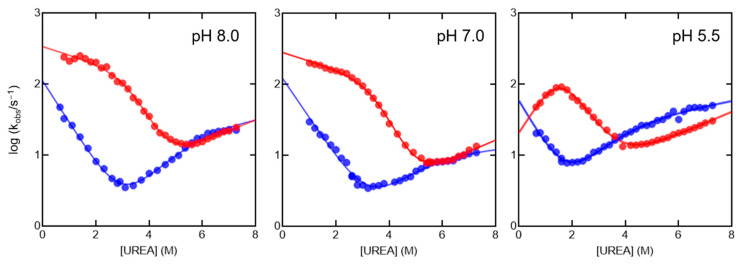
Folding kinetics of the NSH2-CSH2 tandem at different pH conditions. The two phases recorded with different optical filters (320 nm cutoff in red, and 360 nm cutoff in blue) are reported. Interestingly, while the two phases, ascribable to the (un)folding kinetics of the NSH2 and CSH2 domain, resemble what was observed for the isolated domains at pH 8.0 and pH 7.0, lowering the pH to 5.5 causes a slowed down refolding of the NSH2 domain, with increasing kobs, obtained in refolding experiments, at increasing urea concentrations. Kinetic m values for pH 8.0 and 7.0 are the same used for the isolated NSH2 and CSH2 domains (see Figure 3). For pH 5.5 kinetic m values are m_IN_ = 0.80 ± 0.05 kcal mol^−1^ M^−1^; m_DI_ = −1.6 ± 0.1 kcal mol^−1^ M^−1^; m_NI_ = 0.20 ± 0.02 kcal mol^−1^ M^−1^.

It is of interest to compare data obtained for the isolated domains with the folding and unfolding kinetics of the NSH2-CSH2 tandem (Figure 4, Table S2). Curiously, while the two domains display independent folding and unfolding kinetics at pH 8.0 and 7.0, at pH 5.5, the analysis of kinetic data reveals a slowed down refolding. Moreover, it is evident that the *k*_obs_ obtained in refolding experiments at low [urea] increase with increasing denaturant concentrations. This effect has been observed in other multidomain proteins [37,39], and it is compatible with the rapid formation of a misfolded intermediate state, which acts as a bottleneck in the folding pathway, necessitating its unfolding to allow the progression toward the native conformation. Altogether, these results put into evidence an intriguing scenario in which, while at nearly physiological conditions, the NSH2 and CSH2 domains fold and unfold as independent units, shifting towards acidic pH values triggers an intramolecular interaction during the folding process that slows down folding, suggesting a complex dynamic interplay occurring between the two domains.

### 2.3. Exploring the Binding Properties of the NSH2-CSH2 Tandem with Gab2

As described in the introduction, the SH2 domains of SHP2 recognize and bind different ligands in the intracellular environment, triggering the activity of the PTP domain of the protein and the activation/repression of downstream signaling [20,30,40,41,42]. The binding mechanism of the isolated NSH2 and CSH2 domains has been extensively characterized in the recent past. In particular, our group, through a combination of site-directed mutagenesis and kinetic binding experiments, investigated the binding of the two domains with peptides mimicking different portions of Gab2 protein (Grb2 associated binder) [33,34,43]. Here, we resorted to comparing the binding properties of the NSH2-CSH2 tandem of SHP2 with those described for the isolated domains, in order to pinpoint possible changes in affinity and kinetics in the context of the tandem construct.

In analogy to our previous works, we performed kinetic binding experiments by challenging the NSH2-CSH2 tandem with peptides mimicking different portions of Gab2, namely Gab2_608–620_ (binding the NSH2 domain) and Gab2_637–649_ (binding the CSH2 domain). From now on, the two peptides will be named, respectively, Gab2N and Gab2C, while the peptide mimicking the entire segment of Gab2 binding both SH2 domains, and corresponding to Gab2_608–649_, will be named Gab2T. To compare data with our previous works, the experiments were performed in buffer Hepes 50 mM, NaCl 300 mM, 2 mM DTT, pH 7.2, at 10 °C, by following the FRET signal occurring through the excitation of the tryptophan residues in the NSH2-CSH2 tandem with light at 280 nm and following the change in fluorescence of the dansyl group covalently attached to the N-terminus of the peptides.

**Figure 5:**
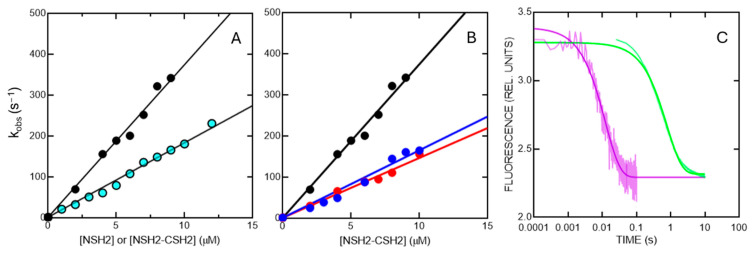
(**A**) Binding kinetics of the NSH2 domain isolated (in light blue) and of the NSH2-CSH2 tandem (in black) versus the Gab2N peptide. It is possible to observe that, in the context of the tandem, the slope of the straight line, which corresponds to the microscopic association rate constant of the reaction, is higher compared to the isolated domain. (**B**) Binding kinetics of the NSH2-CSH2 tandem (in black) versus Gab2N peptide, in the absence of Gab2C (black) and in the presence of 10 µM (in blue) and 20 µM (in red) Gab2C. (**C**) Displacement kinetic traces obtained by rapidly mixing a preincubated complex of NSH2-CSH2 with Gab2N versus an excess of Gab2T (in green) and a preincubated complex of NSH2-CSH2 with Gab2C versus an excess of Gab2T (in purple).

First, to test whether the presence of the contiguous CSH2 affected the affinity of the NSH2 domain for Gab2, we mixed a constant concentration of dansylated Gab2N (1 µM) vs. increasing concentrations of NSH2-CSH2 (ranging from 2 µM to 9 µM). The results are shown in Figure 5A. The obtained observed rate constant (*k*_obs_) as a function of the concentration of the NSH2-CSH2 tandem was fitted with a linear equation, the slope corresponding to the microscopic association rate constant (*k*_on_) and the intercept with the y-axis to the microscopic dissociation rate constant (*k*_off_). To directly measure the *k*_off_, we performed displacement experiments by rapidly mixing a preincubated complex of NSH2-CSH2 with dansylated Gab2N (both at a constant concentration of 2 µM) versus a high excess of non-dansylated Gab2N (50 µM). The analysis of kinetic data shows that in the context of the tandem, the binding of the NSH2 domain with Gab2N peptides occurs with a higher *k*_on_ (and lower K_D_, calculated as *k*_off_/*k*_on_) compared to the isolated domain (respectively 37.3 ± 0.8 µM^−1^ s^−1^ vs. 18.2 ± 0.2 µM^−1^ s^−1^), while the *k*_off_ remains unaffected (1.8 ± 0.1 s^−1^ in both cases). Notably, however, by performing the same experiment in the presence of Gab2C at 10 µM and 20 µM concentrations, we calculated a *k*_on_ of 16.4 ± 0.2 µM^−1^ s^−1^ and 14.4 ± 0.2 µM^−1^ s^−1^, lower compared to what was previously calculated in the absence of Gab2C (37.3 ± 0.8 µM^−1^ s^−1^) (Figure 5B). We performed a binding experiment by rapidly mixing the NSH2 domain with different concentrations of Gab2C peptide, and no kinetic traces could be recorded in the experimental conditions used. Overall, our results are compatible with a scenario in which the presence of the CSH2 domain appears to modulate the binding properties of the NSH2 domain with Gab2, suggesting the presence of an interplay between the two domains in the regulation of the early recognition event.

We also resorted to monitoring the binding reaction between the NSH2-CSH2 tandem with the Gab2T peptide. However, we could not obtain such peptide in an N-terminal dansylated form, so the kinetic binding experiments between the NSH2-CSH2 and Gab2T did not return any change in fluorescence. To obtain information about the ability of the NSH2-CSH2 to bind Gab2T, we performed kinetic displacement experiments in order to test the ability of Gab2T to displace Gab2N and Gab2C. Thus, in two separate experiments, we rapidly mixed a preincubated complex of NSH2-CSH2 with Gab2N (both at a constant concentration of 2 µM) versus an excess of Gab2T (50 µM) and a preincubated complex of NSH2-CSH2 with Gab2C (both at constant concentrations of 2 µM and 10 µM, respectively) versus an excess of Gab2T (50 µM) (Figure 5C). Importantly, in the two experiments, we could calculate two different rate constants, respectively, of 1.2 ± 0.3 s^−1^ in the case of Gab2N, and 82 ± 1 s^−1^ in the case of Gab2C. These two values are in great agreement with what was previously calculated in binding experiments of the isolated domains [34,35]. Thus, our results show that the NSH2 and CSH2 domains, in the context of the tandem, bind, respectively, Gab2N and Gab2C, and that the Gab2T peptide is able to displace both peptides.

## 3. Discussion

Despite considerable efforts to characterize isolated domains regarding their folding mechanisms and binding interactions, a gap remains in understanding these domains within larger supramodular structures. This gap is particularly evident in the context of tandem repeats or multidomain architectures, where little information exists regarding the binding mechanisms of PPI domains. Such an approach is vital for identifying potential dynamic interplays during ligand recognition and binding, such as allosteric and cooperative behaviors, and complex intramolecular energetic networks that may regulate protein function and contribute to cellular signaling networks.

In this work, we provide a comprehensive characterization of the folding mechanism of the NSH2-CSH2 tandem from SHP2 protein, under different pH conditions. Our analysis reveals that the two domains typically fold and unfold independently, while more complex scenarios emerge under acidic pH conditions. However, interactions between the two domains introduce complexities and transient misfolding events, particularly under acidic conditions. This parallels what has been previously observed for the PDZ1-PDZ2 tandem of whirlin [37,38], sPDZD2 [44], and the SH2-CSH3 tandem of Grb2 [39].

Interestingly, the NSH2 and CSH2 domains display a high sequence identity (48.4%). Misfolding events between tandem domains are typically reduced by the evolution through the lowering of sequence identity of adjacent domains, which is generally less than 40% in tandem repeat proteins, while, conversely, high sequence identity may contribute to misfolding events [45,46]. In the case of the NSH2-CSH2 tandem, the two domains behave independently, while misfolding events are triggered by the change in experimental conditions. The underlying mechanisms of this phenomenon for the NSH2-CSH2 construct remain speculative, potentially involving the protonation of specific residues leading to electrostatic interactions causing misfolding and slowing down the process. The possibility of subtle interdomain communication between the SH2 domains of SHP2 protein might be of key importance in regulating the activation of the enzyme [41,47], the NSH2 domain being reported to be a highly dynamic system in isolation [48], and in tandem with the CSH2 domain [32], where the intrinsic structural plasticity of the construct has been proposed to be fundamental for the function of Shp2. In analogy to what has been described for other isolated SH2 domains [43,49], this phenomenon might be due to underlying energetic networks driving domain functions.

Following this, it is of interest to comment on the recent data obtained for the SH2-CSH3 construct of Grb2. In that case, residues at the interface between the two domains are at the basis of a complex energetic network determining the binding specificity of the CSH3 domain [39]. The findings from our study indicate a potential interaction between the NSH2 and CSH2 domains during the binding process with different segments of Gab2. Specifically, there is a major alteration in the microscopic association rate constant of the binding reaction between the NSH2 domain and the Gab2N peptide when the CSH2 domain is already bound to the Gab2C peptide. This result suggests that the recognition of the phosphorylated ligand might occur through a concerted mechanism involving interdomain communication between the two domains, modulating the ability of the NSH2 domain to recognize its partner, and it is in line with previous evidence of the cooperative effect of the tandem SH2 domain in binding bi-phosphorylated ligands and inducing higher activation levels of Shp2 compared to monophosphorylated ligands [25,27]. Further research, employing extensive site-directed mutagenesis, is needed to elucidate the molecular underpinnings of these interdomain interactions with the aim of identifying key residues influencing the folding pathway and the binding mechanism of the NSH2-CSH2 tandem.

## 4. Materials and Methods

### 4.1. Protein Expression and Purification

The constructs encoding the isolated NSH2 domain, the isolated CSH2 domain, and the NSH2-CSH2 tandem were subcloned in a pET28b+ plasmid vector and then transformed in Escherichia coli cells BL21 (DE3). Bacterial cells were grown in LB medium containing 30 μg/mL of kanamycin, at 37 °C until OD_600_ = 0.7–0.8, and then protein expression was induced with 1 mM IPTG. After induction, cells were grown at 37 °C overnight and then collected by centrifugation. The pellet was resuspended in buffer Hepes 50 mM, 300 mM NaCl, pH 7.2, with the addition of an antiprotease tablet (Complete EDTA-free, Roche, Basilea, Switzerland), then sonicated and centrifuged. The soluble fraction from bacterial lysate was loaded onto a nickel-charged Hi-Trap Chelating HP (GE Healthcare, Uppsala, Sweden) column equilibrated with Hepes 50 mM, 300 mM NaCl, pH 7.2. Protein was then eluted with a gradient from 0 to 1 M imidazole by using an AKTA-prime system. Fractions containing the protein were collected and the buffer was exchanged to 50 mM Hepes pH 7.0 by using a HiTrap Desalting column (GE Healthcare). The purity of the protein was analyzed through SDS-page. Peptides mimicking Gab2_608–620_, Gab2_637–649_ and Gab2_608–649_ were purchased from Genscript.

### 4.2. Equilibrium and Kinetic (Un)folding Experiments

Equilibrium unfolding experiments were performed on a Fluoromax single photon counting spectrofluorometer (Jobin-Yvon, Piscataway NJ, USA). All the proteins used were held at a constant concentration of 1 μM, excited at 280 nm and emission spectra were recorded between 300 and 400 nm, at increasing denaturant concentration. Experiments were performed at 25 °C, using a quartz cuvette with a path length of 1 cm. Data from isolated NSH2 and CSH2 domains were analyzed with the following two-state equation

$$y=Y_{N}+Y_{D}\;\frac{e^{\left(m_{D-N}(\left[UREA\right]-{\left[UREA\right]}_{1/2}\right)}}{1+e^{\left(m_{D-N}(\left[UREA\right]-{\left[UREA\right]}_{1/2}\right)}}$$

where m_D-N_ represents the m value for the unfolding reaction, and [UREA]_1/2_ is the midpoint of the transition. The curve obtained from the NSH2-CSH2 tandem was fitted to the sum of two two-state equations.

Rapid mixing for kinetics folding and unfolding experiments were carried out on a stopped-flow device (Pi-star, Applied Photophysics, Leatherhead, UK) and fluorescence emissions of NSH2, CSH2 and NSH2-CSH2 tandem were measured with 320 nm cutoff filter and a 360 nm cutoff filter using an excitation wavelength of 280 nm (see Section 2 for details). The kinetic folding experiments were made with a 1 μM final concentration of protein and urea as a denaturing agent. The temperature was set at 25 °C. Buffers used were Sodium Acetate 50 mM pH 5.5, Hepes 50 mM pH 7.0, Tris HCl 50 mM pH 8.0. For each experiment conducted, an average of 5 independent kinetic traces was fitted with a single exponential decay. Chevron plots were fitted to equations implying the accumulation of an intermediate in rapid pre-equilibrium with the denatured state [35]

$$k_{obs}=\frac{k_{IN}^{0}\exp\left(-m_{IN}\left[UREA\right]/RT\right)}{\left(1+K_{DI}exp(m_{DI}\left[UREA\right]/RT\right)}+k_{NI}^{0}exp(m_{NI}\left[UREA\right]/RT)$$

and to equation a assuming the absence of populated low energy intermediate(s) and ascribing the curvature on the unfolding arm to a change in the rate-limiting step at different denaturant concentrations [34].

$$k_{obs}=k_{f}^{0}exp(-m_{f}[UREA]/RT)+\frac{k_{u}^{0}exp(m_{u}[UREA]/RT)}{1+K_{part}exp(m_{part}[UREA]/RT)}$$

### 4.3. Kinetic Binding Experiment

Kinetic experiments of binding between the NSH2, CSH2 and NSH2-CSH2 tandem and the peptides mimicking different portions of Gab2 were measured by monitoring FRET between the tryptophan, acting as a donor, and a dansyl group covalently attached to the N-terminus of the peptides, acting as acceptor. The experiments were performed on a single-mixing SX-18 stopped-flow instrument (Applied Photophysics, Leatherhead, UK) recording the change of dansyl emission. The excitation wavelength used was 280 nm while the fluorescence emission was collected using a 475 nm cutoff glass filter. The binding experiments were carried out at 25 °C mixing a constant concentration of dansylated peptide (1 μM) versus increasing concentrations of NSH2, CSH2 and NSH2-CSH2 (ranging from 2 to 10 μM). The buffer used was Hepes 50 mM, NaCl 300 mM, pH 7.2. The observed rate constants were calculated from the average of 3–6 single traces and by fitting the time-course for binding using a single exponential equation

$$y=A\exp\left(-k_{obs}\;t\right)+c$$

where *A* is the amplitude of the kinetic trace, *k*_obs_ the observed rate constant and *c* the final fluorescence value of the trace. The dependences of *k*_obs_ as a function of ligand concentrations were fitted with a linear equation

$$k_{obs}=k_{on\;}\left[ligand\right]+k_{off}$$

where *k*_on_ is the microscopic association rate constant, and *k*_off_ the microscopic dissociation rate constant.

## Data Availability

The original contributions presented in the study are included in the article and Supplementary Material; further inquiries can be directed to the corresponding author.

## Supplementary Materials

The following supporting information can be downloaded at: https://www.mdpi.com/article/10.3390/ijms25126566/s1.
