# Supplementary material for: Folding and Binding Kinetics of the Tandem of SH2 Domains from SHP2
Source: Int J Mol Sci. 2024 Jun 14;25(12):6566. doi: 10.3390/ijms25126566 (PMC11203950; doi:10.3390/ijms25126566)
Supplement: Supplementary file 1 [file ijms-25-06566-s001.zip › ijms-3039525-supplementary.pdf]

Isolated NSH2	k_{IN} (s^{-1})	k_{NI} (s^{-1})	K_{DI}
**pH 5.0**	450 ± 10	5.1 ± 0.3	0.004 ± 0.001
**pH 5.5**	360 ± 10	2.5 ± 0.2	0.004 ± 0.001
**pH 7.0**	950 ± 30	2.0 ± 0.3	0.003 ± 0.001
**pH 7.5**	960 ± 30	2.4 ± 0.2	0.005 ± 0.001
**pH 8.0**	800 ± 40	3.1 ± 0.3	0.003 ± 0.001
**pH 8.5**	850 ± 30	3.8 ± 0.4	0.004 ± 0.001

Isolated CSH2	k_f (s^{-1})	k_u (s^{-1})	k_{part}
**pH 5.0**	41 ± 4	3.1 ± 0.5	0.004 ± 0.001
**pH 5.5**	126 ± 6	0.54 ± 0.11	0.004 ± 0.001
**pH 7.0**	510 ± 20	0.05 ± 0.02	0.003 ± 0.001
**pH 7.5**	590 ± 30	0.04 ± 0.01	0.005 ± 0.001
**pH 8.0**	510 ± 20	0.06 ± 0.02	0.003 ± 0.001
**pH 8.5**	360 ± 20	0.05 ± 0.02	0.004 ± 0.001

Table S1 – Kinetic parameters calculated at different pH conditions for the isolated NSH2 and CSH2 domain.

Tandem NSH2-CSH2	k_{IN} (s^{-1})	k_{NI} (s^{-1})	K_{DI}	k_f (s^{-1})	k_u (s^{-1})	k_{part}
**pH 5.5**	100 ± 10	2.2 ± 0.3	60 ± 6	60 ± 5	0.84 ± 0.08	0.004 ± 0.001
**pH 7.0**	280 ± 10	0.9 ± 0.1	0.003 ± 0.001	120 ± 5	0.11 ± 0.02	0.003 ± 0.001
**pH 8.0**	330 ± 10	1.9 ± 0.3	0.003 ± 0.001	350 ± 30	0.04 ± 0.02	0.002 ± 0.001

Table S2 - Kinetic parameters calculated at different pH conditions for the tandem NSH2-CSH2 domain. k_{IN}, k_{NI} and K_{DI} parameters refer to the phase observed with 320nm cutoff filter, while k_f, k_u and k_{part} parameters refer to the phase observed with the 360nm cutoff filter.

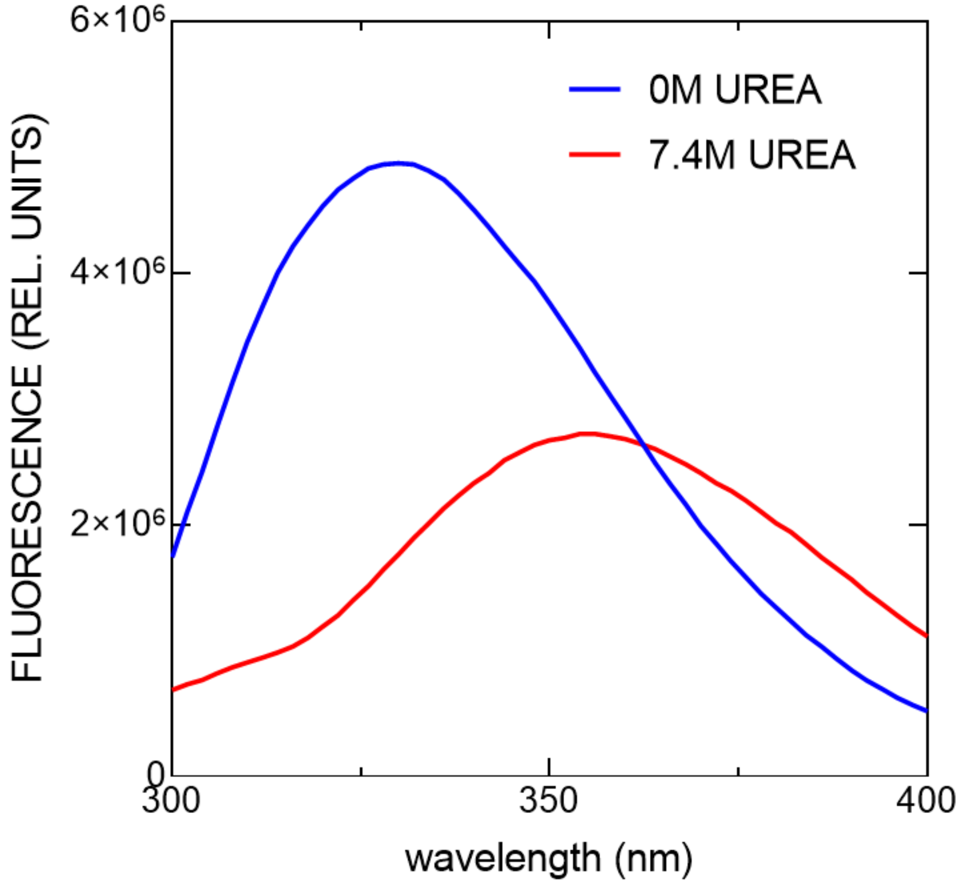

Figure S1 – Comparison of fluorescence emissions of the NSH2-CSH2 tandem construct at 0M urea (in blue) and at 7.4M urea (in red). It is possible to observe a decrease in fluorescence emission between the native and denatured protein, as well as a peak shift towards higher wavelength upon denaturation.
